# The epigenetic regulators EP300/CREBBP represent promising therapeutic targets in MLL-rearranged acute myeloid leukemia

**DOI:** 10.1038/s41420-024-01940-5

**Published:** 2024-05-01

**Authors:** Wenqi Wu, Yanan Jiang, Donghui Xing, Yixin Zhai, Huimeng Sun, Xiang He, Kaiping Luo, Pengpeng Xu, Feng Pan, Guolei Dong, Guibing Ren, Zhigang Zhao

**Affiliations:** 1 Department of Senior ward, Tianjin Medical University Cancer Institute and Hospital, National Clinical Research Center for Cancer, Key Laboratory of Cancer Prevention and Therapy, Tianjin’s Clinical Research Center for Cancer, Tianjin, 300060 China; 2 Department of Medical Oncology, Tianjin First Central Hospital, School of Medicine. Nankai University, Tianjin, 300192 China; 3 Department of Oncology, Characteristic Medical Center of Chinese People’s Armed Police Force, Tianjin, 300162 China; 4 Department of Molecular Medicine, the University of Texas Health Science Center at San Antonio, San Antonio, TX 78229-3904 USA; 5 Department of Breast Oncology, National Clinical Research Center for Cancer, Key Laboratory of Cancer Prevention and Therapy, Tianjin’s Clinical Research Center for Cancer, Key Laboratory of Breast Cancer Prevention and Therapy, Tianjin Medical University Cancer Institute and Hospital, Tianjin, 300060 China

**Keywords:** Acute myeloid leukaemia, Targeted therapies

## Abstract

Acute myeloid leukemia (AML) with mixed-lineage leukemia (*MLL*) gene rearrangements (MLL-r) is an aggressive subtype of blood cancer with dismal prognosis, underscoring the urgent need for novel therapeutic strategies. E1A-binding protein (EP300) and CREB-binding protein (CREBBP) function as essential transcriptional coactivators and acetyltransferases, governing leukemogenesis through diverse mechanisms. Targeting EP300/CREBBP holds great promise for treating leukemia with some certain cytogenetic abnormalities. Here, we demonstrated that EP300 and CREBBP are core epigenetic regulators in the pathogenesis of MLL-r AML through assaying the transposase-accessible chromatin with high-throughput sequencing (ATAC-seq). Knocking-out *EP300/CREBBP* and inhibitor (A-485) treatment depressed the MLL-r cells proliferation, while the MLL wild-type cells remained uninfluenced. We found that the CDK4/RB/E2F axis was downregulated specifically in MLL-r AML cell after A-485 treatment by RNA-seq, western blot and cut-tag analyses. EP300/CREBBP inhibitor selectively exerted potent anti-leukemia activity through blocking the MLL-r-BET complex binding to H3K27Ac modification on critical genes loci, distinct from global histone acetylation. Collectively, our study identified EP300/CREBBP as a critical epigenetic driver of MLL-r leukemia and validated their therapeutic potential through targeting inhibition, offering a promising avenue for improving clinical outcomes in this aggressive leukemia.

## Introduction

The *MLL* gene on chromosome 11q23 is disrupted in a unique group of AMLs, with a prevalence of approximately 10% of all adults AML cases [1]. The MLL-r results in chimeric MLL-fusion proteins, comprising of the N-terminus from MLL protein and the C-terminus from one of over 120 known fusion partners [2, 3]. This cytogenetic heterogeneity fosters diverse leukemic phenotypes and contributes to the dismal prognosis, despite current therapeutic options [3, 4].

MLL-r proteins exert their oncogenic potential in hematopoietic stem cells by driving transcriptional dysregulation of target genes (*HOXA9*, *MEIS1* and *PBX3*) [5]. The aberrant transcriptional program is mediated through chromatin modifications that promote malignant transformation, but imparting targetable therapeutic dependencies [6–8]. Recent study found that MLL fusion proteins recruit histone methyltransferase DOT1L containing transcription elongation complex to specific oncogenic transcription factors, facilitating gene activation and leukemia progression [9]. Other studies further highlighted the critical role of chromatin accessibility and its related genes in the MLL-r AML pathogenesis and prognosis, providing an opportunity for therapeutics intervention [5, 10].

*EP300* and its homologue *CREBBP* encode highly related acetyltransferases that act as transcriptional coactivators in multiple signaling pathways [11, 12], suggesting the potential involvement of *EP300/CREBBP* in cancers [13]. Notably, *EP300/CREBBP* participate in transcriptional regulation and chromatin modifications for the establishment of transcriptional regulation system, with a major role in maintaining normal hematopoiesis as well as promoting leukemogenesis [14, 15]. Recent evidences further implicated their aberrant function in the development of multiple hematologic malignancies [16]. Importantly, targeting the bromodomain of EP300/CREBBP has shown promising efficacy in several malignancies driven by transcriptional activator-driven malignancies, including leukemia [17], lymphoma [18] and myeloma [19]. However, the precise role of *EP300*/*CREBBP* in MLL-r leukemia remains largely unexplored. In this study, we investigated the dependence of *EP300/CREBBP* in MLL-r AML and evaluated the anti-tumor efficacy of EP300/CREBBP inhibitor. We found that chromatin accessibility controls underlying fundamental properties of MLL-r leukemia cells during the process of AML initiation and progressions. Moreover, we provided a proof of concept that targeting EP300/CREBBP is a potential therapeutic for MLL-r AML, as validated by in vitro and in vivo leukemia models. Mechanistic studies revealed the selective EP300/CREBBP inhibitor disrupting the binding of MLL-r-BET transcriptional complex to H3K27Ac modification on some certain critical gene loci to inhibit MLL-r AML cells proliferation, independent of their global histone acetylation status.

## Results

### Accessible chromatin and transcriptome landscapes analyses identified the regulatory role of EP300/CREBBP in MLL-r leukemia

**Fig. 1:**
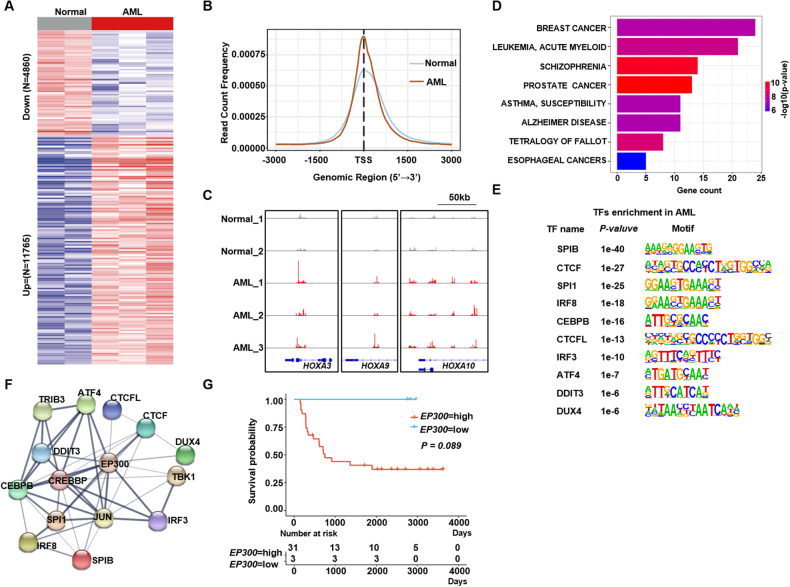
EP300/CREBBP is the core transcription factor in the MLL-r AML. **A** Heatmap of different ATAC-seq peaks among AML and control cells. **B** Average signal of chromatin accessibilities across the promoter region (TSS-3kB/+3kB) of a random set of expressed genes. **C** Gene tracks of the ATAC-seq signal at HOXA gene family. **D** Bar plots of diseases analyzed by enriched peaks of ATAC-seq in AML cells. **E** Top 10 motifs and their binding TFs in AML cells analyzed by Homer. **F** PPI network of the enriched TFs in AML cells. **G** Kaplan-Meier plots of the MLL-r AML cohort (*n* = 34) of *EP300* low and high expression (optimal cut-off: 13.31741) groups of TARGET cohort (https://portal.gdc.cancer.gov). Control: CD34^+^ cord blood cells; AML: MLL-AF9 driving AML.

Using previously established MLL-AF9 leukemia model that inducing umbilical cord blood CD34^+^ cells develop into AML cells by MLL-AF9 fusion gene [20, 21]. We detailed the transcriptional and chromatin dynamics of control umbilical cord blood CD34^+^ cells and MLL-r AML cells. ATAC-seq revealed distinct open chromatin regions and associated gene expression changes between the two type cells. Analysis of the ATAC-seq data uncovered 16,625 different-count peaks (DCPs) in MLL-r AML cells compared to CD34^+^ controls, indicating the major differences in chromatin accessibility (Fig. 1A). Notably, MLL-r AML specific DCPs were enriched within proximal promoters (-3kb/+3 kb) (Fig. 1B), highlighting potential transcriptional influence. A snapshot of the ATAC-seq profiles showed that there were more accessible peaks at known MLL-r target genes (*HOXA3, HOXA9* and *HOXA10*) in MLL-r AML cells compared to CD34^+^ control cells (Fig. 1C). Genes associated with DCPs exhibited a highly significant cancer specific signature including AML, breast cancer and prostate cancer (Fig. 1D).

To link changes in accessible chromatin with specific transcription factors (TFs), HOMER motif analysis of open chromatin regions enriched in MLL-r AML cells was used. This identified top 10 TFs (Fig. 1E) involving in hematopoietic cells proliferation and differentiation (*SPIB, ATF4*) [22, 23] and pathogenesis of leukemia (*CEBPB*, *CTCF*) [24, 25]. Most of these TFs are the independent prognostic factors for AML patients [24–26]. To further investigate their potential functional interactions, we employed STRING analysis that predicts protein-protein interactions [27]. An interacted network derived from these 10 factors was identified, with the EP300 and CREBBP positioned at the core site (Fig. 1F). Moreover, the clinical AML data from the Target dataset showed that high level of *EP300* expression correlated with significant inferior overall survival time (OS) in MLL-r AML patients (Fig. 1G). We then analyzed the selective dependency on *EP300* and *CREBBP* of a series of leukemia compared to other tumor cells in DepMap database, highlighting *EP300/CREBBP* potential targeting in AML cells (Additional file 1: Figure S1A). In clinical data of the Target cohort, AML patients with higher *EP300* or *CREBBP* expression exhibited strikingly inferior OS than those with lower *EP300* or *CREBBP* expression (Additional file 1: Fig. S1B). Although ATAC-seq results demonstrated that *EP300/CREBBP* had a critical role in MLL-r AML development, the expression of *EP300*/*CREBBP* were upregulated in MLL-r cells as well as in AML cells with other genetic mutations (Additional file 1: Fig. S1C), suggesting *EP300*/*CREBBP* function were not close to their expression. In addition, previous reports had showed interactions between EZH2 and MYB with EP300 in some genotypes AMLs, presenting therapeutic targeting also [14, 28, 29]. However, the function and underlying mechanism of *EP300/CREBBP* in MLL-r AML remain uncharacterized, we will uncover it detailly in this study.

### *EP300/CREBBP* are required for MLL-r leukemia cells proliferation and stemness maintenance

**Fig. 2:**
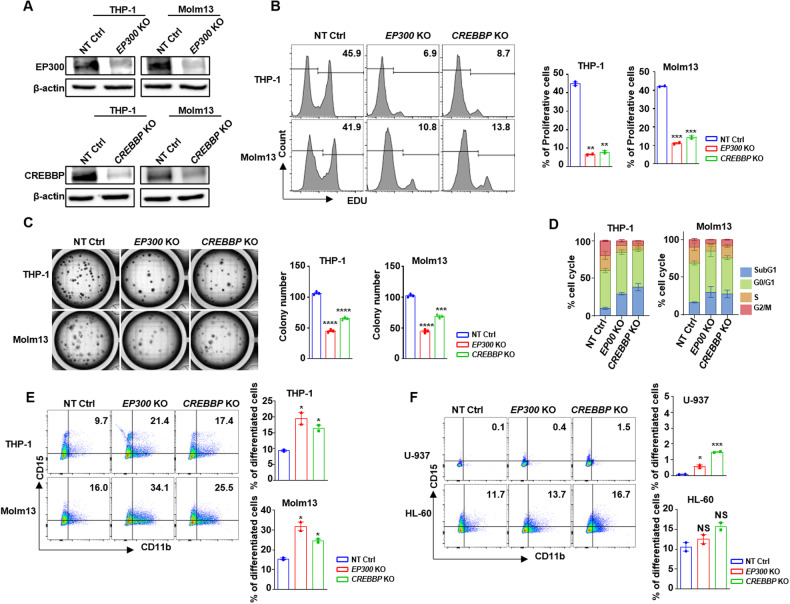
Knocking out *EP300* and *CREBBP* inhibited proliferation of MLL-r cells. **A** Western blots of cells knocking out *EP300*, *CREBBP* and NT Ctrl cells. **B** Proliferation analysis with EdU staining of cells knocking out *EP300*, *CREBBP* and NT Ctrl. **C** Quantification of colony forming in *EP300*, *CREBBP* knock-out and NT Ctrl cells. **D** The changes of cell cycle after knocking out *EP300* and *CREBBP*. **E**, **F** The proportion of CD11b^+^CD15^+^ cells with *EP300*, *CREBBP* knocked out and NT Ctrl in MLL-r (**E**) and MLL-r wild-type (**F**) cells. NT Ctrl Nontarget Control, KO Knock Out, MLL-r MLL rearrangement, MLL-r cell lines THP-1, Molm13, MLL wild-type cell lines: U-937, HL-60. NS: No Significance.

To explore the roles of *EP300* and *CREBBP* in MLL-r AML cells, *EP300* or *CREBBP* was knocked out respectively by CRISPR-Cas9 mediated sgRNA targeting (Fig. 2A and Additional file 1: Fig. S2A) in both MLL-r and MLL wild-type cell lines. We found that the proliferation and colony formation functions of MLL-r AML cells were suppressed significantly upon loss of *EP300* or *CREBBP* (Fig. 2B, C), while, the proliferation of the MLL wild-type cells was less influenced, highlighting a specific vulnerability of MLL-r leukemia to EP300/CREBBP depletion (Additional file 1: Fig. S2B). Moreover, the percentage of MLL-r AML cells at G0/G1 phase increased, accompanied by a marked decrease in S and G2/M phases after loss of *EP300* or *CREBBP* (Fig. 2D). We also observed a marked differentiation of MLL-r cells as evidenced by an increase of CD11b^+^CD15^+^ cells, following knock-out (KO) *EP300* or *CREBBP* (Fig. 2E). Consistent with this, Wright-Giemsa staining indicated enhanced differentiation morphology in MLL-r cells upon *EP300/CREBBP* loss compared to MLL wild-type cells. These findings suggested that KO *EP300* or *CREBBP* promoted differentiation and potentially reduced proliferation in MLL-r AML cells but not MLL wild-type cells (Fig. 2F and additional file 1: Fig. S2C). Furthermore, to elucidate the molecular mechanism underpinning these observations, RNA-seq analysis was performed following *EP300* KO in MLL-r as well as MLL wild-type cells. We found that the proliferation-associated genes were significantly down-regulated including *E2F1* gene family, and *MCM2* gene family, whereas these genes were remained largely unaffected in MLL wild-type AML cell, further emphasizing the differential dependence of *EP300* (Additional file 1: Fig. S2D). Gene set enrichment analysis (GSEA) revealed cell division-associated pathways including E2F-targets, G2M-checkpoint and MYC-targets were depressed in MLL-r cell after KO *EP300* (Additional file 1: Fig. S3A). In contrast, the downregulated pathways in MLL wild-type cell after KO *EP300* such as hypoxia, myogenesis and estrogen-response, were not associated with cell growth (Additional file 1: Fig. S3B). These findings collectively indicated that the MLL-r AML cells exhibited a robust dependence on *EP300/CREBBP* for essential proliferative and stemness-maintaining, distinct from MLL wild-type cells.

To determine the functional synergy between *EP300* and *CREBBP* in MLL-r AML cells, *EP300* and *CREBBP* were deleted simultaneously. H3K27Ac is the major chromatin modification mediated by EP300 and CREBBP. We found that loss of *EP300* and/or *CREBBP* in MLL-r AML cells led to significant decrease in global histone H3K27Ac intensity (Additional file 1: Fig. S3C). No statistically significant differences were observed in colony formation in MLL-r AML cells between single *EP300* KO and combined EP300/*CREBBP* KO. Whereas, combined *EP300*/*CREBBP* KO exhibiting a marked inhibition of colony formation compared to only *CREBBP* KO (Additional file 1: Fig. S3D and S3E). However, combined *EP300*/*CREBBP* KO inhibited the proliferation more significant than single gene KO in MLL-r AML cells (Additional file 1: Fig. S3F). These findings indicated that *EP300* and *CREBBP* played cooperative roles in regulating leukemia cell growth, whereas the *EP300* had more critical function than *CREBBP* in maintaining MLL-r AML cell stemness.

### EP300/CREBBP inhibitor exhibited high potential in MLL-r AML

**Fig. 3:**
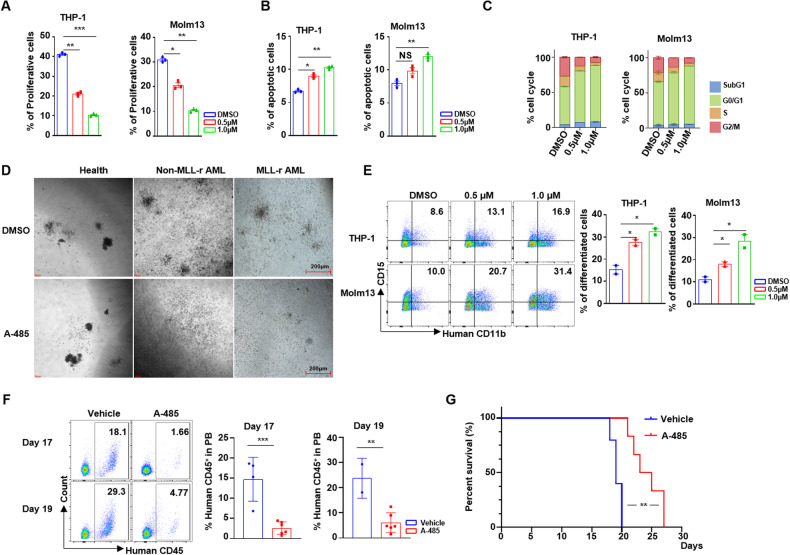
A-485 selectively targeting HAT domains of EP300/CREBBP suppressed proliferation of the MLL-r cell lines. **A** Proliferation analysis by EdU staining of MLL-r cell lines treated with A-485 or DMSO. **B** The bar plots of apoptosis of MLL-r cell lines treatment with A-485 or DMSO. **C** Cell cycle profile of MLL-r cell lines after treating with A-485 or DMSO. **D** Colony morphology with primary MNCs from healthy donor, MLL wild-type and MLL-r AML patient treatment with A-485 or DMSO. **E** The expression of CD11b^+^CD15^+^ in MLL-r cells treated with A-485. **F** Percentage of human CD45^+^ leukemia cells in peripheral of NSG transplanted with THP-1 cell line and treated with A-485 or vehicle for 14 Days. **G** Kaplan-Meier plots of the OS time of AML mice administered with A-485 or vehicle. MLL-r MLL rearrangement, MLL-r cell lines: THP-1, Molm13; MLL wild-type cell lines: U-937, HL-60. NS No Significance.

Our KO results identified *EP300/CREBBP* as a potential therapeutic target in MLL-r AML. A recent study reported A-485, a new inhibitor selectively targeting histone acetyltransferase (HAT) domain of EP300/CREBBP, showed antitumor effects in several hematologic malignancies and prostate cancer [13]. We subsequently investigated the therapeutic potential of A-485 in MLL-r AML cells. Treatment of MLL-r cells with A-485 resulted in reduced proliferation in a concentration-dependent manner, but A-485 exhibited moderated apoptotic induction (Fig. 3A, B). Consistent with our genetic studies, the percentage of MLL-r AML cells in G0/G1 phase increased, while S and G2/M phases decreased significantly after A-485 treatment (Fig. 3C). By contrast, MLL wild-type AML cells (HL-60 and U-937) were substantially resistant to A-485 (Additional file 1: Fig. S4A) with no significant changes in proliferation, apoptosis and cell cycle upon treatment (Additional file 1: Fig. S4B–D). Furthermore, the colony formation from MLL-r AML patient primary bone marrow cells was inhibited after A-485 treatment compared to DMSO control. Intriguingly, A-485 exhibited minimal impact on colony forming in MLL wild-type AML patient primary cell and healthy hematopoietic cells (Fig. 3D). Previous study reported that the deletion of *EP300* promoted the cancer stem cell differentiation [30]. We observed a marked increase in the proportion of CD11b^+^CD15^+^ cells, which demonstrates more mature neutrophil was increased in MLL-r cells than MLL wild-type AML cells after A-485 treatment (Fig. 3E and Additional file 1: Fig. S4E). Similarly, the morphology of the A-485 treated cells showed more band neutrocyte than DMSO treated cells by Wright-Giemsa dye staining in MLL-r AML cells but not MLL wild-type AML cells (Additional file 2). Taken together, our data demonstrated that A-485 not only inhibited the proliferation of MLL-r AML cells, but also induced their differentiation.

We then assessed the potential therapeutic effect of A-485 in vivo using two established MLL-r AML xenograft models. We first transplanted THP-1 cell line (MLL-r) into the NSG mice then treated them with A-485 or vehicle control. A-485 administration significantly decreased the engraftment of human CD45^+^ leukemic cells in the peripheral blood compared to the control group (Fig. 3F). Moreover, A-485 treatment significantly extended OS time (Fig. 3G), suggesting its efficacy in eradicating MLL-r AML cells in vivo. We further evaluated A-485 in a usually used mouse AML model derived from mouse hematopoietic stem cells and progenitor cells infected with the lentivirus carrying the *MLL-AF9* fusion gene. Consistent with the THP-1 mouse model, mice treated with A-485 exhibited significantly lower leukemia burden in peripheral blood compared to the control group (Additional file 1: Fig. S4F). Importantly, A-485 was well-tolerated, with no significant difference in body weight observed between the treatment and control groups (Additional file 1: Fig. S4G). A-485 treatment resulted in a marked increase in overall survival times (Additional file 1: Fig. S4H). These findings demonstrated that A-485 significantly attenuated the MLL-r AML burden in vivo, while exhibiting minimal side-effects on normal hematopoietic cells.

### A-485 down-regulated the CDK4/RB/E2F pathway to suppress proliferation of MLL-r AML cells

**Fig. 4:**
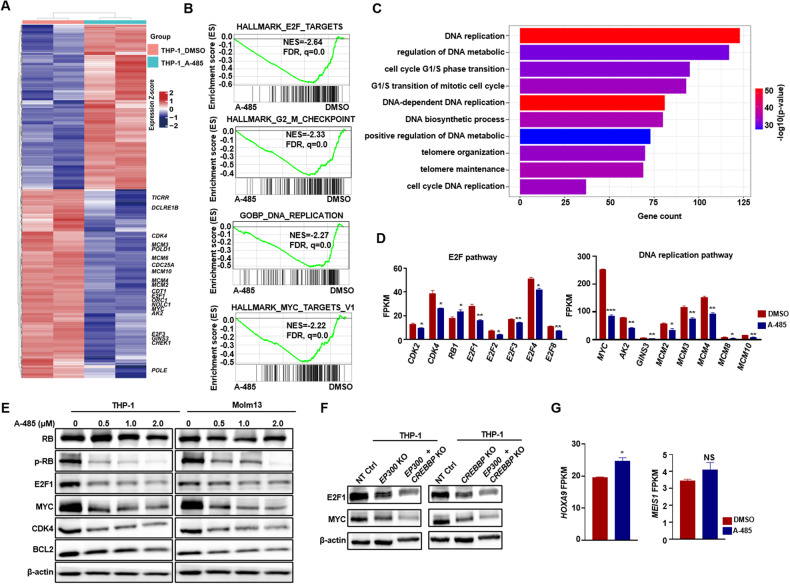
A-485 depressed CDK/RB/E2F pathway associated genes expressing in MLL-r cells. **A** Heatmap of differentially expressed genes between A-485 and DMSO treatment in MLL-r cells (THP-1). The marked genes are critical to proliferation. **B** GSEA analysis using differentially expressed genes in MLL-r cells treatment with A-485 or DMSO. **C** Bar plots of biological process (BP) pathways from GO database enriched by down-regulated genes from MLL-r cells, treated with A-485 compared to DMSO. **D** Validation of the expression of genes related to the E2F (left) and DNA replication (right) pathways from MLL-r cell line treated with A-485 or DMSO. **E** The western blot of the CDK/RB/E2F pathway-associated proteins in MLL-r cell lines treated with different dose of A-485. **F** The western blot of E2F1 and MYC in MLL-r cells knocked out EP300 and CREBBP. **G** mRNA expression of *HOXA9* and *MEIS1* of MLL-r cells after A-485 and DMSO treatment. MLL-r cell lines: THP-1 and Molm13. NS: No Significance.

To explore the molecular mechanisms underlying the sensitivity of MLL-r AML cells to A-485, we performed RNA-seq in both MLL-r and MLL wild-type AML cells with DMSO or A-485 treatment. Compared to DMSO treatment, A-485 treatment resulted in substantial transcriptional alterations in MLL-r cells, with 2428 genes upregulated (*p* < 0.05) and 2787 genes downregulated (*p* < 0.05). Downregulated genes in MLL-r cells were enriched in pathways associated with cell proliferation, cell cycle and DNA replication, while no such gene was observed in MLL wild-type cells treated with A-485 (Fig. 4A; Additional file 1: Fig. S5A). There were a few of genes commonly downregulated in MLL-r and MLL wild-type cells after A-485 treatment, moreover these genes were not related to cell proliferation or differentiation (Additional file 1: Fig. S5B). GSEA revealed the E2F pathway, G2M pathway, DNA replication and MYC targets were decreased markedly in the MLL-r AML cells after A-485 treatment (Fig. 4B), but these changes were not observed in the MLL wild type cells (Additional file 1: Fig. S5C). The E2F family of transcription factors reportedly promotes cell proliferation, regulates cell cycle, and facilitates DNA replication through multiple mechanisms [31]. Consistently, gene ontology (GO) analysis also revealed that gene sets associated with cell proliferation, DNA replication and cell cycle were significantly depressed in MLL-r AML cells after A-485 treatment but not in MLL wild-type AML cells (Fig. 4C; Additional file 1: Fig. S5D). In addition, genes in E2F pathway and DNA replication pathway were significantly downregulated after A-485 treatment (Fig. 4D). We also confirmed that the expression of proteins related with E2F pathway (CDK4, p-RB, E2F1, BCL2) were downregulated in MLL-r AML cell after A-485 treatment (Fig. 4E). Furthermore, downregulations of E2F were also validated by western blot in MLL-r AML cells with knocking-out of *EP300* or *CREBBP* (Fig. 4F). As previous report the CDK4-RB-E2F pathway is a targeting therapeutic in many tumors [32, 33], we found the CDK4-RB-E2F pathway genes including CDK4, E2F1, E2F3 and BCL2, were inhibited after A-485 treating in MLL-r cells. We then examined the expression of *Hoxa9* and *Meis1*, known MLL target genes, but neither *HOXA9* nor *MEIS1* was altered after A-485 treatment (Fig. 4G). These findings suggested that the A-485 function the antileukemia effects through inhibiting the CDK4/RB/E2F pathway, but independent of the classical MLL-HOXA9 signaling pathway in MLL-r AML.

### A-485 played anti-leukemia function through disrupting the MLL-r-BET transcriptional complex binding to H3K27Ac

**Fig. 5:**
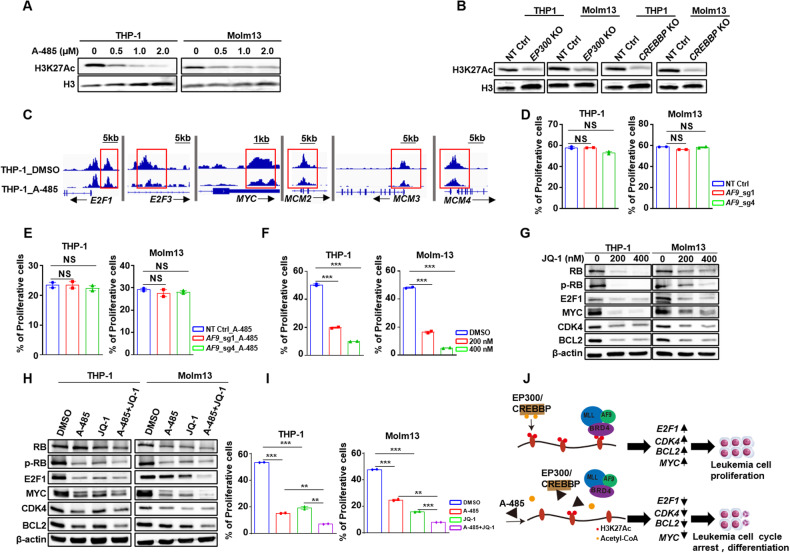
A-485 suppresses MLL-r-BET complex attaching to the H3K27Ac to down-regulate CDK/RB/E2F-pathway. **A** The western blot of H3K27Ac and H3 modification of MLL-r cell lines treated with different dose of A-485. **B** The western blot of H3K27Ac of MLL-r cell lines after knocking out *EP300* and *CREBBP*. **C** Gene tracks of H3K27Ac signals in E2F-pathway-related genes regions of THP-1 treated with A-485 and DMSO. **D** The bar plot of the proliferative cells in MLL-r cell lines after knocking out *AF9*. **E** The proliferation of MLL-r cell lines knocking out *AF9* treatment with A-485. **F** Bar plot of proliferative cells of MLL-r cell lines treatment with JQ-1. **G** Western blot of the E2F-pathway-related proteins in MLL-r cell lines treated with different concentration of JQ-1. **H** Western blot of E2F-pathway-related proteins in MLL-r cell lines treated with DMSO, A-485, JQ1 and combination of A-485 and JQ-1. **I** The proliferation of MLL-r cell lines treated with DMSO, A-485, JQ-1 and combined A-485 and JQ-1. **J** Model of how A-485 to inhibit proliferation of MLL-r cell. MLL-r cell lines including THP-1 and Molm13. NS: No Significance.

H3K27Ac promotes chromatin accessibility and transcriptional activation [34]. EP300/CREBBP preferentially acetylates histone H3K27 [16], while A-485 effectively reduced H3K27Ac in solid tumors [12, 35]. Consistent with prior studies, A-485 treatment reduced H3K27Ac level in both MLL-r and MLL wild-type AML cells (Fig. 5A Additional file 1: Fig. S6A), mirroring the observation of *EP300* or *CREBBP* deletion (Fig. 5B, Additional file 1: Fig. S6B). To analyze A-485-mediated changes in H3K27Ac, we performed CUT-Tag with H3K27Ac specific antibody and coupled with high-throughput sequencing in AML cells treatment with A-485 or DMSO. The enrichment of H3K27Ac were reduced at the promoter regions of *E2F1*, *E2F3*, *MCM2*, *MCM3* and *MCM4* in MLL-r AML cells after A-485 treatment, but the modification of H3K27Ac remained unaltered in MLL wild-type AML cells, (Fig. 5C, Additional file 1: Fig. S6C). These findings suggested that A-485 downregulates key genes transcription in MLL-r cells by specifically reducing H3K27Ac modification at these gene loci. While A-485 treatment and *EP300* or *CREBBP* KO also significantly reduced the global histone modification of H3K27Ac in MLL wild-type AML cells, the genes related to E2F, DNA replication and cell proliferation were uninfluenced (Additional file 1: Fig. S6D). The protein level of MYC was depressed in both genotype cells, whereas, no cell-proliferation inhibition was observed in MLL wild-type cells, which demonstrated the growth of MLL wild-type AML cells is minimally required of MYC. These observations further underscored the selective targeting of A-485 in MLL-r cells.

Previous study has highlighted the AF9 protein has the ability of binding to the histone acetylation loci [36]. Therefore, we examined whether the anti-leukemia activity of A-485 requires targeting the AF9-acetylation interaction. *AF9* knock-out had minimal impact on leukemia cell growth (Fig. 5D). Moreover, there was no difference in proliferation between control and AF9-deficient MLL-r AML cells after A-485 treatment (Fig. 5E). These data suggested that targeting the AF9-acetylation interaction is not essential for the anti-leukemia effect of A-485 in MLL-r AML cells.

MLL-r protein combined with BET family proteins forming a super transcriptional complex that utilizes bromodomain to bind H3K27Ac [17, 37]. We used the BET inhibitor (JQ-1) to block the transcriptional complex binding to H3K27Ac and found its pronounced ability to inhibit MLL-r cell proliferation (Fig. 5F). Similar to A485 treatment, JQ-1 downregulated the expression of the key proteins related with E2F and DNA replication (Fig. 5G). Notably, the combination of A-485 and JQ-1 synergistically reduced the protein expression compared to single-agent treatment (Fig. 5H). Simultaneous targeting of EP300/CREBBP and BET had synergistic anti-leukemia effects in MLL-r AML (Fig. 5I). Taken together, our data revealed that A-485 exerted its anti-leukemic function in MLL-r AML through reducing H3K27Ac at the key gene loci, thereby disrupting the binding of MLL-r-BET complex to H3K27Ac (Fig. 5J) and downregulated the expression of proliferation-related genes.

## Discussion

The complex interplay between epigenetic regulators and MLL fusion proteins drives the pathogenesis of AML [5]. Our study shed light on the crucial role of *EP300/CREBBP* in the MLL-r leukemogenesis and identifies their potential as therapeutic targets. Our findings revealed that both EP300 and CREBBP function as essential coactivators for MLL-r-mediated gene expression, promoting proliferation and stemness maintenance of MLL-r AML cells. This was in line with previous observations that the oncogene roles of *EP300/CREBBP* in MLL-r AML leukemogenesis, as evidenced by MLL-CREBBP fusions [38]. However, the distinct functions of these coactivators in different hematological malignancies remain an intriguing area for further investigation.

Our research underscored the synergistic effect of EP300 and CREBBP in regulating MLL-r leukemia cell proliferation. Both co-activators demonstrated common and distinct roles, suggesting that simultaneous inhibition could yield synergistic therapeutic effects. Our observations found that EP300/CREBBP selectively inhibitor A-485 exerted a robust eradicating leukemia effect in MLL-r AML cells. Importantly, this effect was specific to MLL-r cells, exhibiting minimal impact on MLL wild-type AML cell and normal hematopoiesis, and highlighting the potential of EP300/CREBBP as selective therapeutic targets for this aggressive leukemia subtype of MLL-r.

Mechanistically, A-485 treatment led to a significant decrease in H3K27Ac levels, a hallmark of EP300/CREBBP activity. However, global histone H3K27Ac reduction was not selective to MLL-r cells, suggesting that the anti-leukemic effect was not solely dependent on global histone acetylation. Notably, A-485 significantly altered the expression of key genes involved in cell cycle progression and DNA replication specifically in MLL-r cells. This suggested that A-485 disrupted the MLL-r-dependent transcriptional program minimally utilizing the lost global histone acetylation. Further investigation of the molecular mechanisms underlying this selective targeting is warranted.

Several lines of evidence pointed the importance of the CDK4/RB/E2F axis in leukemogenesis [31, 39]. Our data revealed that the key proteins related with CDK4/RB/E2F axis were downregulated in MLL-r AML cell after A-485 treatment, potentially contributing to the anti-proliferative effects. This finding aligned with the changes in signaling pathways related to AML progression, cell proliferation, DNA replication and metastasis, which provided explains of molecular mechanisms for its anti-tumor efficacy in MLL-r AML.

The MLL-AF9 fusion protein interacts with BET family proteins (BRD3/4), forming a transcriptional complex that binds to H3K27Ac on chromatin [17]. Furthermore, the combination of A-485 and JQ-1 exhibited synergistic anti-leukemic effects, suggesting a potential strategy for combinatorial therapy in MLL-r AML. Both A-485 and JQ-1 downregulated expression of key genes associated with cell cycle progression and DNA replication (E2F1, BCL2, CDK4 and c-MYC), likely by disrupting the binding of these transcriptional complexes to chromatin. This finding indicated the importance of targeting MLL-r associated transcriptional complexes as a therapeutic strategy for MLL-r AML.

Our study established EP300/CREBBP as targetable dependencies in MLL-r AML. EP300/CREBBP inhibitor exhibited promising therapeutic potential by disrupting MLL-r-BET transcript complex binding to H3K27Ac on key gene loci distinct from global histone acetylation, and disrupting critical signaling pathways in MLL-r AML. The observed paved the way for further investigation of therapy strategies. Future work will aim to define the molecular relationship between EP300/CREBBP and the MLL-r-BET complex to further refine and optimize targeted therapies for MLL-r AML.

## Materials and Methods

### Cell lines and patient cohort

Cell lines were acquired from American Type Culture Collection (ATCC). THP-1 (MLL-AF9), Molm13 (MLL-AF9), U-937 and HL-60 were maintained in RPMI-1640 medium (Gibco) supplemented with 10% fetal bovine serum (NEWZERUM) and 1% pen/strep (Gibco) at 37 °C and 5% CO_2_. The 293 T cell was cultured in DMEM (Gibco) supplemented with 10% fetal bovine serum and 1% pen/strep at 37 °C and 5% CO_2_.

These cell lines were negative for mycoplasma through recently testing by the PCR Mycoplasma Test Kit (HUABIO, K0103) and were authenticated by short-tandem repeat (STR) analysis.

The gene-expression and clinical information of TARGET cohort patients (*n* = 156) including MLL-r subgroup (*n* = 34) were download from https://portal.gdc.cancer.gov. The dependence of *EP300* and *CREBBP* data (*n* = 929) were obtained from the DepMap data base (https://depmap.org/portal/). The BEAT AML database (http://www.vizome.org/aml2/geneset/) only has gene-expression data of AML patient (*n* = 134). These data have similar variance and obey the normal distribution.

### Cell viability assay

The AML Cells were plated in 96-well plates, 3 × 10^^5^ cells/ml, 100 μl/well, treated with limiting dilutions of inhibitors, incubated for 72 h. Viabilities of cells were detected by CCK8 (ZETA LIFE) according to the instruction, then to detect the value of optical density (OD). Inhibitor was purchased from MedChemExpress (MCE).

### Gene knocking-out

The clustered regularly interspaced short palindromic repeats (CRISPR) mediated sgRNAs targeting *EP300*, *CREBBP* and *AF9* were cloned into lentiviral vector (Addgene, 52961) [40], which was digested by restriction enzyme BsmBI-V2 (NEB, R0739). The lentivirus was produced by 293 T cells, which were transfected with psPAX2 (Addgene), pMD2.G (Addgene), lentiviral plasmid and polyethylenimine (Polysciences, 24765-1). Virus was collected at 72 h then to infect cells. These cells for analyzing proliferation and western blot were collected after infecting 96 h. The sgRNA sequences as follow: sg_*EP300*: CACCGATTAAAAATGGCCGAGAATG, sg_*CREBBP*: CACCGAGCGGCTCTAGTATCAACCC, non-target_sg: ACGGAGGCTAAGCGTCGCAA; sg_*AF9*_1: GATGGTGTTCGTACGCGGTC; sg_*AF9*_4: AAGTTAGCTTTTCACAGCGG.

### Cell growth, differentiation and apoptosis analysis

For cell growth analysis, the cells were fixed the with 4% paraformaldehyde (Solarbio, P1110) for 15 min at room temperature, then permeated with 0.3% Triton-X (Solarbio, 9002-93-1) for 15 min at room temperature. Next the cells were stained with EdU according to the instruction of EdU kit (Beyotime, C0071L), and analyzed the percentage of the EdU positive cells by flow cytometer (BD Canto II). The cell cycles were performed following the instructions of the cell cycle kit (Beyotime, C1052).

To analyze the differentiation of cell, the cells were incubated with anti-human CD11b, CD15 antibody (BioLegend) at 4 °C for 30 min then washed once with PBS (Gibco). The proportion of CD11b^+^ CD15^+^ cells (differentiated myeloid cell) [41] was detected by flow cytometer. On the other hand, for morphology analysis, we spined these cells to slide and stained them with Wright-Giemsa dye, then to observe under the microscope (Leica).

Cells apoptosis was tested by Annexin-V Apoptosis Detection Kit (BD, 559763). Generally, cells were mixed with 100ul binding buffer supplemented with 1ul Annexin-V PE and 1ul 7-AAD incubating at 4 °C for 30 min, then to analyze by the flow cytometer.

ALL results of flow cytometer were analyzed by FlowJo V10.

### RNA-seq and Cut-Tag analysis

We treated THP-1 and HL-60 cells with DMSO or A-485 for 24 h, then collecting those cells to extract total RNA. Total amounts and integrity of RNA were assessed using the RNA Nano 6000 Assay Kit of the Bioanalyzer 2100 system (Agilent Technologies, CA, USA). The library of mRNA was obtained by the NEBNEXT Ultra Directional RNA Library Prep Kit for Illumina [42] and sequenced with pairing end of 150 bp using the Illumina NovaSeq 6000.

We performed quality control of the RNA-seq data and mapped them to the reference genome of hg38 by the Hisat2. The different expressed genes were analyzed by the DESeq2 R package (1.20.0), and the significant different genes were obtained under the criteria of *p*-value < 0.05 (supplementary table 1). Heatmap was performed by the pheatmap (v1.0.12) R package. Gene Ontology (GO) pathways were analyzed by the clusterProfiler (v3.18.1) R package using the significantly down-regulated genes. GSEA were performed with all differentially expressed genes using the GSEA software (v4.1.0).

We performed the Cut & Tag (Vazyme, TD903) analysis using the antibody targeting the H3K27Ac using the THP-1 and HL-60 cell lines treatment with A-485 or DMSO for 24 h. For Cut&Tag sequencing, we performed the high throughput sequence by the Illumina NovaSeq 6000. The data were filtered by the trim Galore (v0.4.2) and aligned to the refence genome of hg38 using Hisat2 (v2.2.1). We used the MACS2 (v2.1.1) to call peaks, and annotated the peaks by R package of ChipSeeker [43]. We found the Motifs by Homer and analyzed the PPI network using the STRING (https://string-db.org/). The interested peaks were visualized by IGV tools (v2.9.4). These analyses were referred to the instructions of R packages.

### Western blot

For western blot analysis, AML cells were lysed by the RIPA buffer (Solarbio, R0010) supplemented with protease inhibitor (Roche), phosphatase inhibitor (Solarbio, P1260) and PMSF (Beyotime, ST506). Proteins were quantified by the BCA kit (Beyotime, P0012S). Protein samples were separated by SDS-PAGE and then were transferred onto PVDF membranes (Millipore, IPVH00010). The membranes were blocked by 5% skim milk (BD, 232100) at room temperature for 1 h, then incubated with primary antibody at 4 °C overnight. Next, the secondary antibody linked with HRP was incubated at room temperature for 1 h. Then used the chemiluminescent HRP substrate (Millipore, P90719) to detect the signal. The antibodies of western blot were used including EP300 (CST: 54062 S), CREBBP (CST, 7349 S), β-actin (Proteintech, 81115-1-RR), RB (Proteintech), p-RB (Abcam, ab184796), c-MYC (CST: 5605 T), E2F1 (CST, 3742 S), CDK4 (Proteintech, 11026-1-AP), BCL-2 (CST, 4223 T), H3K27Ac (CST, 8173 S), H3 (CST, 4499 S), Casps3 (ABMART, T4044F) and PARP (ABMART, T40050). The full and uncropped western blots are in the Supplemental Material.

### Colony assay

For colony analysis, the cell lines of knocking-out *EP300* or *CREBBP* were cultured in semisolid media (StemCell Technologies, H4100), 750 cells/ml, 500 ul/per-well in 24-well plate, then incubated in 5% CO_2_ and 37 °C for 8–10 days to count numbers. We scanned the colonies by CYTATION imaging reader (BioTek).

For human sample, the mononuclear cells (MNCs) were collected from AML patients (1 patient with MLL-r, 1 patient with MLL wild-type) and healthy donor (1 donor), who were given the informed consent of Declaration of Helsinki and approved by the Ethical Committee of Tianjin Medical University Cancer Institute and Hospital. We performed density gradient centrifugation to get MNCs from total bone marrow using lymphocyte separation medium (TBD, LTS1077). Then, the MNCs were seeded on H4434 semisolid media (StemCell Technologies, H4434) by 4 × 10^^4^ cells/ml, 500 ul/ per well of 24-well plate, which were treated with DMSO and A-485, and incubated in 5% CO_2_ and 37 °C. Colonies were enumerated after 8–10 days.

### In Vivo treatment

Animal studies and experimental protocols were approved by the Animal Ethical and Welfare Committee of Tianjin Medical University Cancer Institute and Hospital. These mice were purchased from SPF (Beijing Biotechology) and housed in specifical pathogen free (SPF) environment of no more than 5 mice per cage in Tianjin Medical University Cancer Institute and Hospital Animal Center. We transplanted THP-1 cell line into NSG (NOD-Prkdcscid-Il2rgeml) mice (female, 8 weeks old, near 20 g/mouse) via tail vein with 2 ×10^^5^ cells/mouse. Treatment was start at 3 days after transplantation and continuing for 14 days. These mice were randomly divided into two groups and 7 mice each group. The A-485 was administrated intraperitoneally at a dose of 100 mg/kg once daily. A-485 was dissolved in 5% DMSO (Solarbio), 40% PEG300 (Sigma), 5% tween-80 (Solarbio) and 50% normal saline. After two weeks, the leukemia cells were detectable, and we detected the circulating leukemia cells every two days using 10 ul blood from the tail vein, staining with anti-human CD45^+^ antibody (BioLegend). Meanwhile, we transplanted the mouse AML cells derived from the MLL-AF9 into c57bl/6 mouse (female, 8 weeks old, near 20 g/mouse) through tail vein. These mice were randomly divided into two groups and 7 mice each group treatment with A-485 or Vehicle, then analyzed the leukemia progression as the NSG AML model.

### Statistical analysis

All cell experiments were replicated more than two times and obviously abnormal value according to the variation analysis was excluded. Unpaired two-tailed Student’s t test was used to analyze the percentage of proliferation, apoptosis, cell of differentiation, colony number, human CD45^+^ cells, and expression of mRNA. The mean value is used as the center value and the standard deviation (SD) represents the error bars in bar plot. The different of overall survival time was calculated by the Kaplan-Meier analysis with the log-rank (Mantel-Cox) test. Statistical significance was defined as *P < 0.05; **P < 0.01; ***P < 0.001; ****P < 0.0001.

### Supplementary information

Supplementary materials

Supplemntary Table1 Different expression genes of RNA-seq

Full and uncropped western blot

## Data Availability

All data used in this study are available in this article and supplementary files.
