# Supplementary material for: The epigenetic regulators EP300/CREBBP represent promising therapeutic targets in MLL-rearranged acute myeloid leukemia
Source: Cell Death Discov. 2024 May 1;10:206. doi: 10.1038/s41420-024-01940-5 (PMC11063202; doi:10.1038/s41420-024-01940-5)
Supplement: Supplementary file 1 — Supplementary materials [file 41420_2024_1940_MOESM1_ESM.pdf]

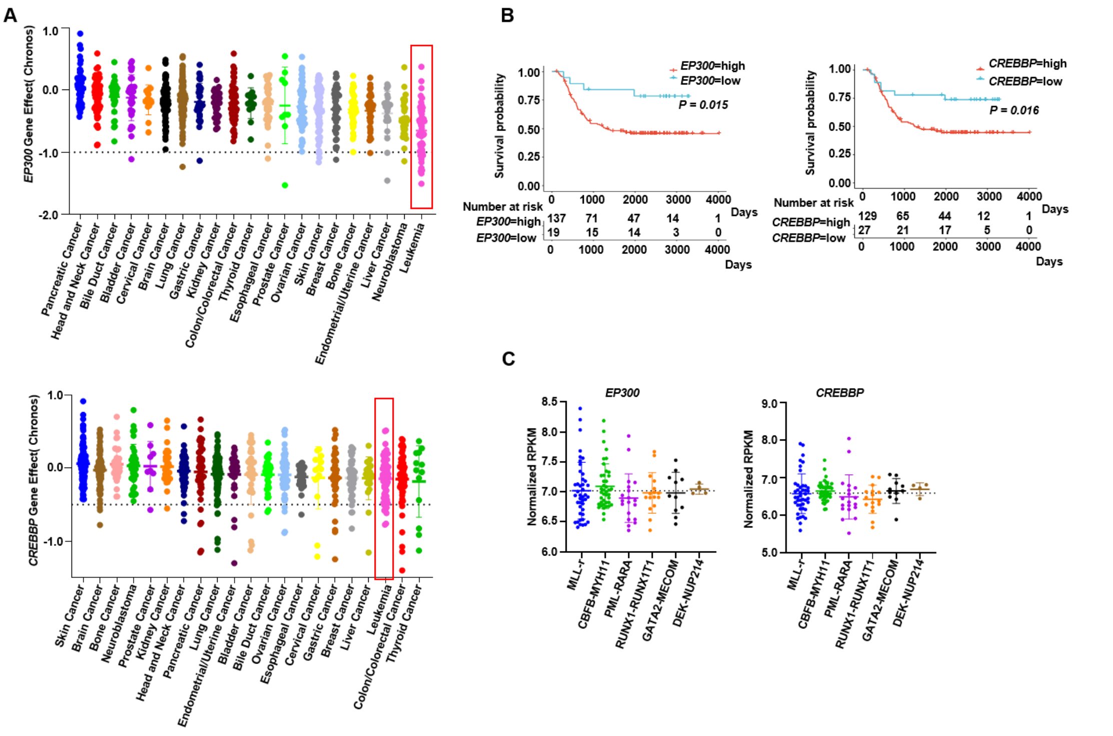

Figure S1. The high expression of the *EP300* and *CREBBP* are risk factor of AML patients.

(A) The dot plots of dependency on *EP300* (top) and *CREBBP* (bottom) in a range of tumors cell lines from the DepMap 22Q2 dataset. (B) Kaplan-Meier plots in Target AML cohort (n=156) of *EP300* expression (optimal cut-off: 13.33901) and *CREBBP* expression (optimal cut-off: 13.27132). (C) The expression of EP300 and CREBBP of different genotypes subgroups from the Beat AML datasets (n=134).

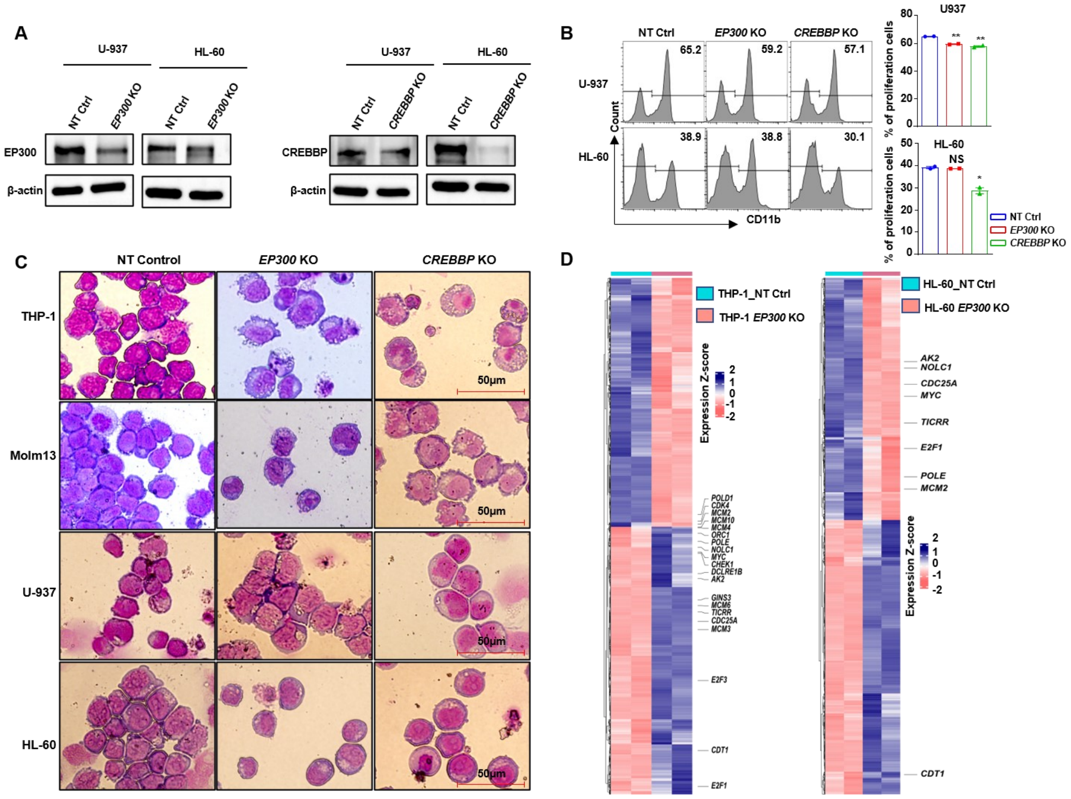

Figure S2. Knocking-out *EP300* and *CREBBP* had less influence in MLL wild-type cells.

(A) The western blot of EP300, CREBBP after KO EP300 and CREBBP in MLL wild-type cells. (B) Proliferation analysis by EdU staining in MLL wild-type cells after KO *EP300* or *CREBBP*. (C) Morphology of MLL-r and MLL wild-type cells after KO *EP300* and *CREBBP*. (D) The significantly different genes in MLL-r and MLL wild-type after KO EP300 or CREBBP. NT Ctrl: Nontarget Control; KO: knocking-out; MLL-r cell: THP-1, Molm13, MLL wild-type: U-937, HL-60.

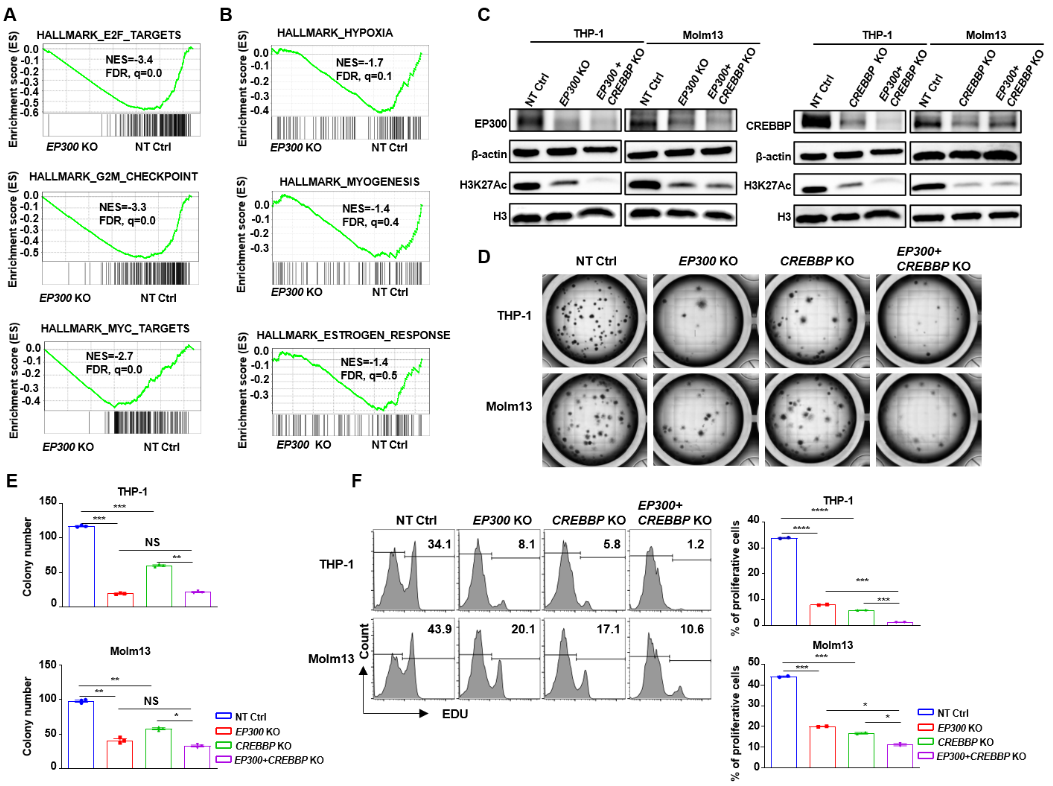

Figure S3 Simultaneously knocking out *EP300* and *CREBBP* inhibited MLL-r cells growth synergistically.

(A-B) GSEA analyzed the noticeable different pathways between *EP300* KO and control cells in MLL-r (A) and MLL wild-type cells (B). (C) Western blot of EP300, CREBBP and H3K27Ac after both KO *EP300* and *CREBBP*. (D-E) Colony forming analysis of EP300 and CREBBP simultaneously KO cells. (F) The proliferation of cells after KO *EP300* combining *CREBBP* analyzed by the EdU staining.

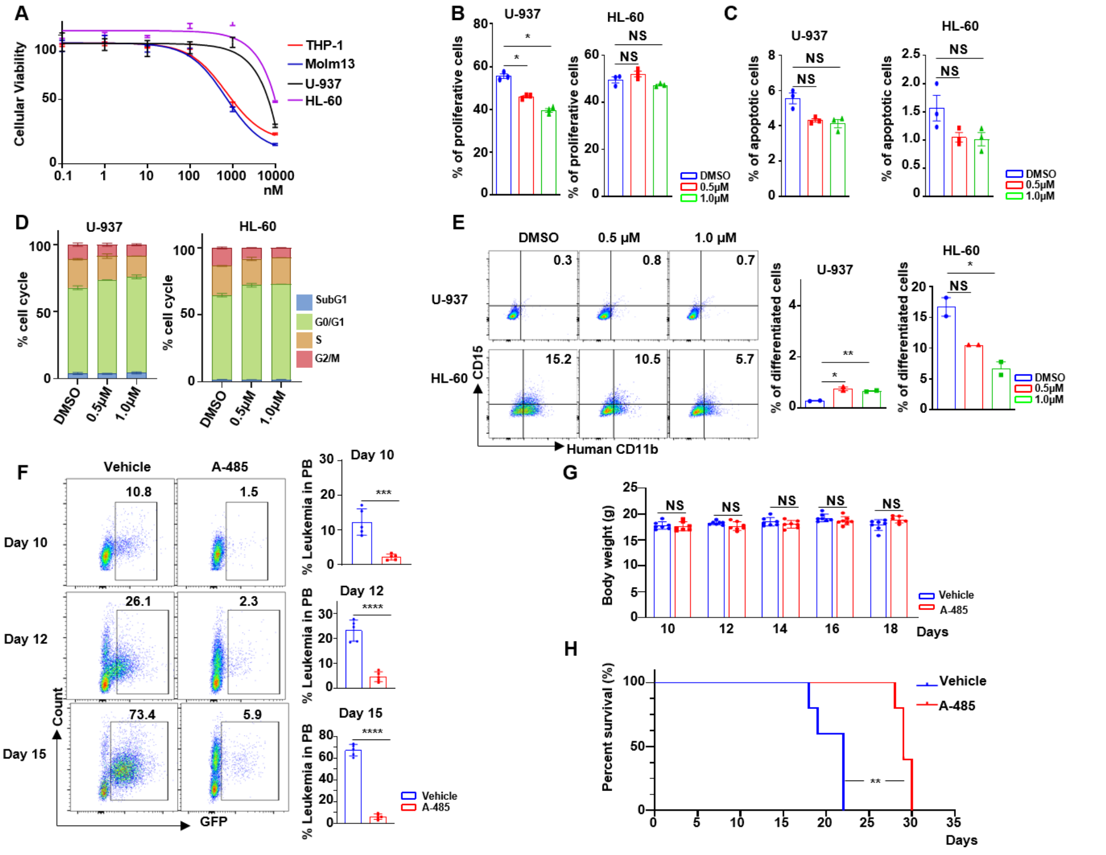

Figure S4. MLL wild-type cell lines had less response to A-485.
(A)A-485 IC50 concentrations in AML cell lines. (B) Proliferation analysis by EdU staining of MLL wild-type cell lines treated with A-485 and DMSO. (C) Bar plot of apoptotic cells of MLL wild-type cells treated with A-485 and DMSO. (D) Cell cycle of MLL wild-type cell lines after treatment with A-485 and DMSO. (E) Differentiated cells ($CD11b^{+}CD15^{+}$) after A-485 treating of MLL wild-type cells. (F) Percentage of leukemia cells in peripheral blood of MLL-AF9 derived AML mice. (G) Body weight difference between A-485 and vehicle treatment. (H) Mice survival time after treating A-485 and vehicle. MLL-r cell: THP-1, Molm13, MLL wild-type: U-937, HL-60.

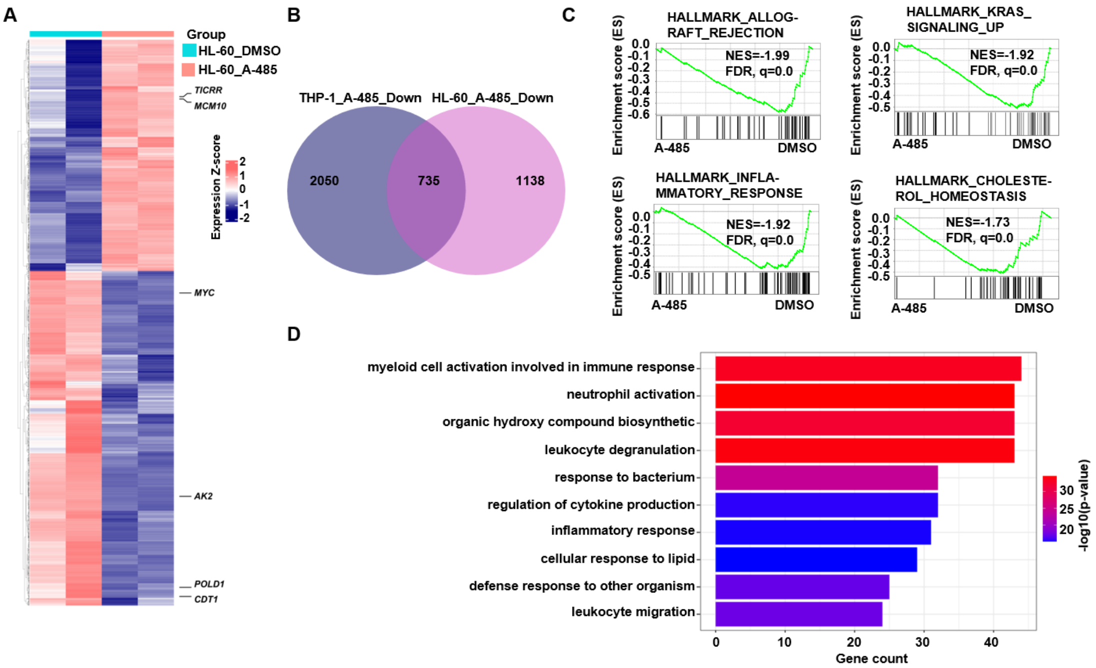

Figure S5. The profile of gene expression in MLL wild-type cells treatment with A-485.

(A) The heatmap of differentially expressed genes ($p<0.05$) in MLL wild-type cells (HL-60) treated with A-485 compared to DMSO.(B) The Venn diagram of overlapped genes between THP-1 and HL-60 cell lines down-regulated by A-485.(C) GSEA of the down-regulated pathways in MLL wild-type cells treatment with A-485 compared to DMSO.(D) Bar plots of BP pathways from GO data base analyzed by the down-regulated genes in MLL wild-type cells treatment with A-485 compared with DMSO.THP-1: MLL-r cell lines, HL-60: MLL wild-type cell lines.

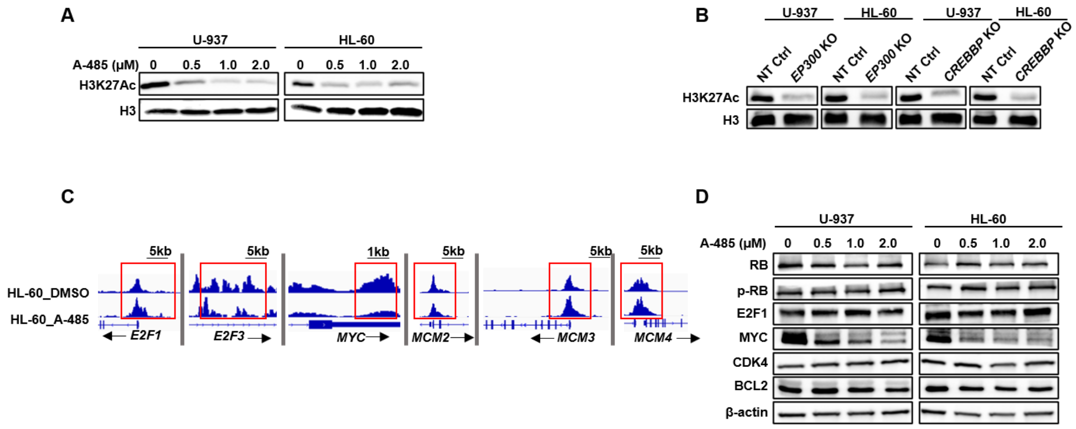

Figure S6 MLL wild-type cells treated with A-485.

(A) The western blot of H3K27Ac and H3 modification of MLL wild-type cell lines treated with different dose of A-485. (B) The western blot of H3K27Ac in MLL wild-type cell lines after knocking out *EP300* and *CREBBP*. (C) Gene tracks of H3K27Ac signals in E2F-pathway-related genes regions of HL-60 cells treated with A-485 and DMSO. (D) Western blot of E2F-pathway-associated proteins in MLL wild-type cell lines treated with different concentration of A-485. MLL wild-type cell lines including U-937 and HL-60.

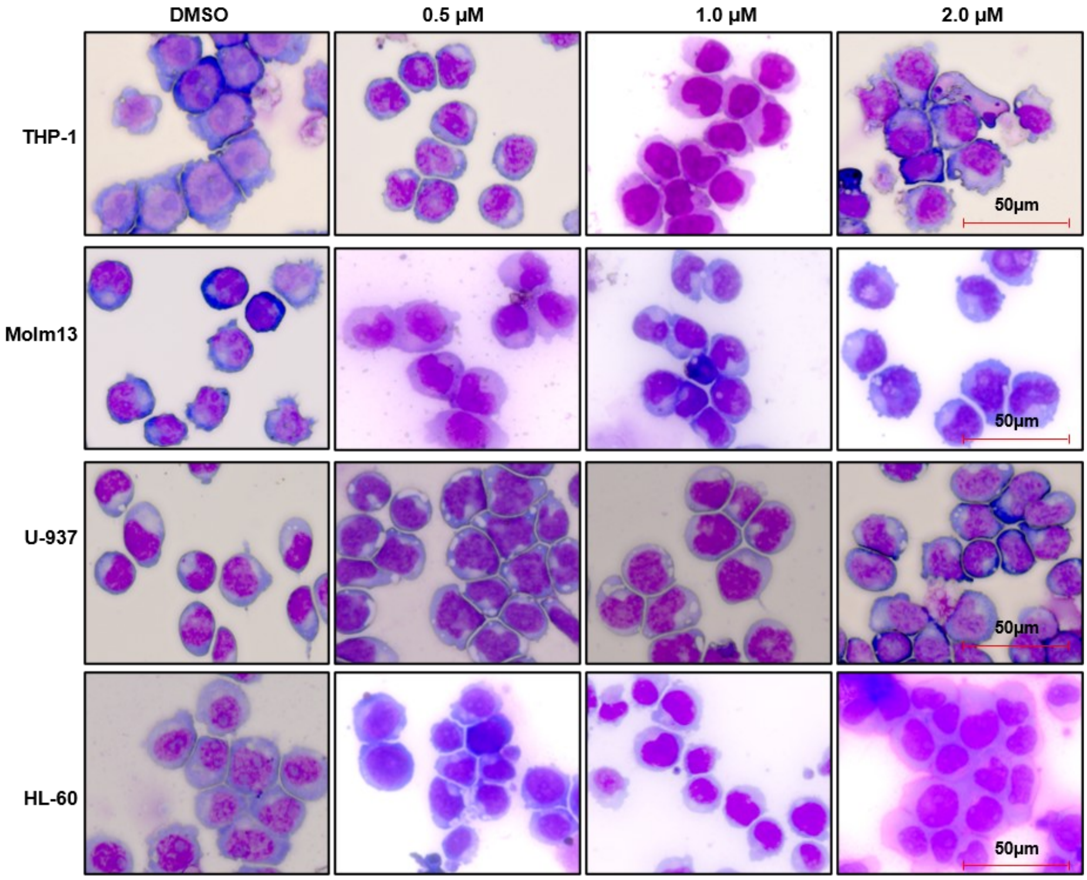

Additional file 2 Morphology changes of AML cells after treatment with A-485.
