# Supplementary material for: The epigenetic regulators EP300/CREBBP represent promising therapeutic targets in MLL-rearranged acute myeloid leukemia
Source: Cell Death Discov. 2024 May 1;10:206. doi: 10.1038/s41420-024-01940-5 (PMC11063202; doi:10.1038/s41420-024-01940-5)

Full and uncropped western blot for Figure 2A

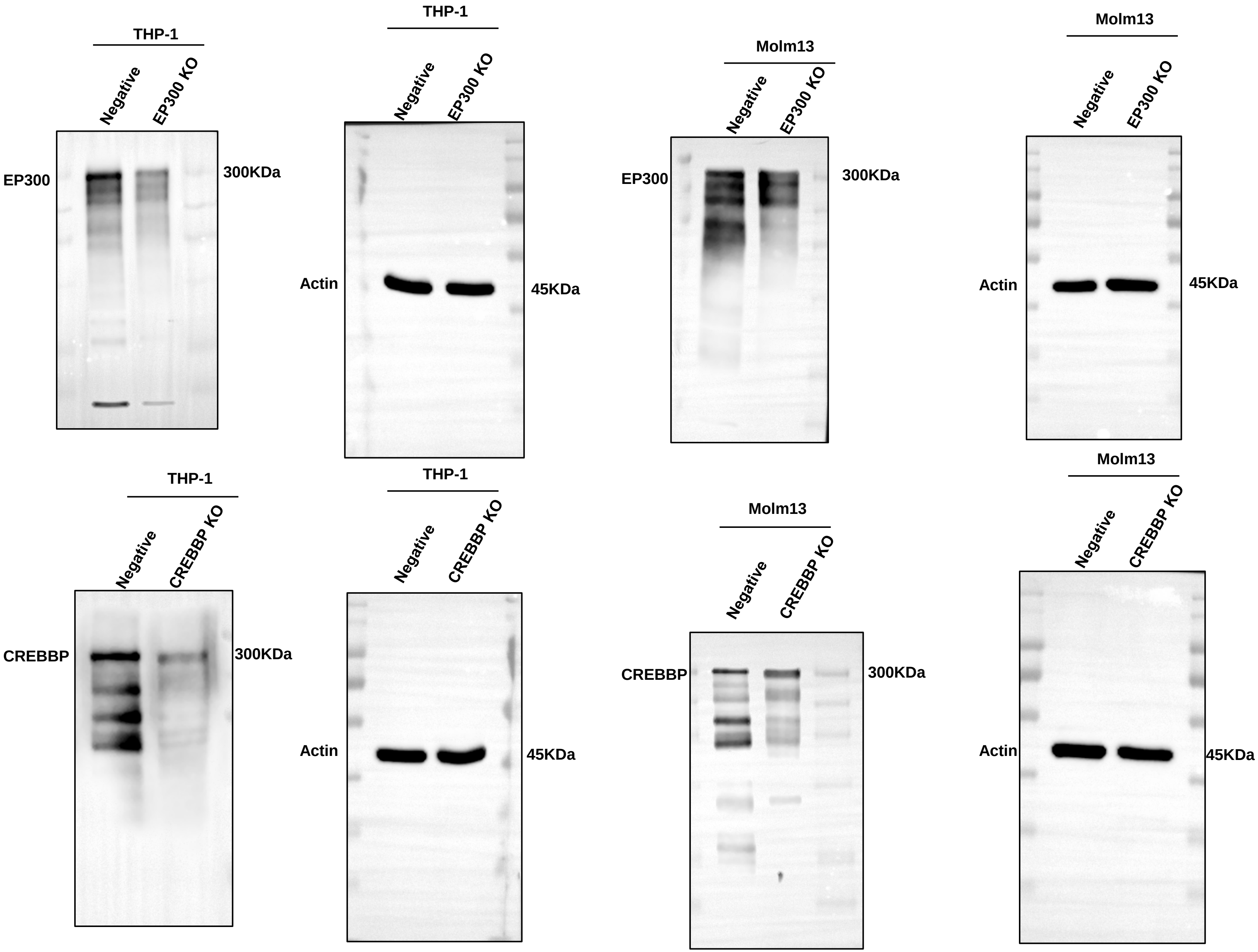

Full and uncropped western blot for Figure S2A

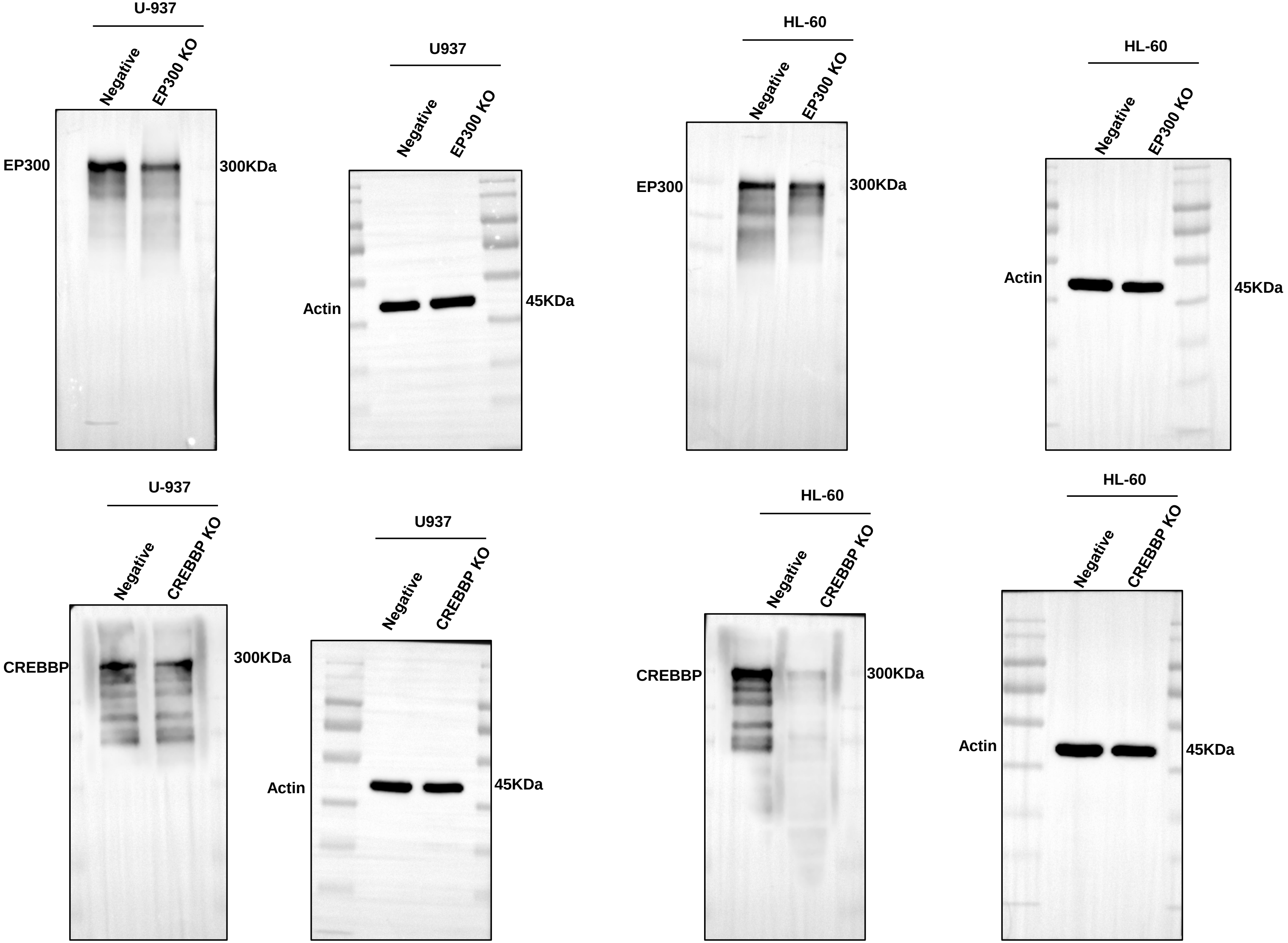

Full and uncropped western blot for Figure 4E

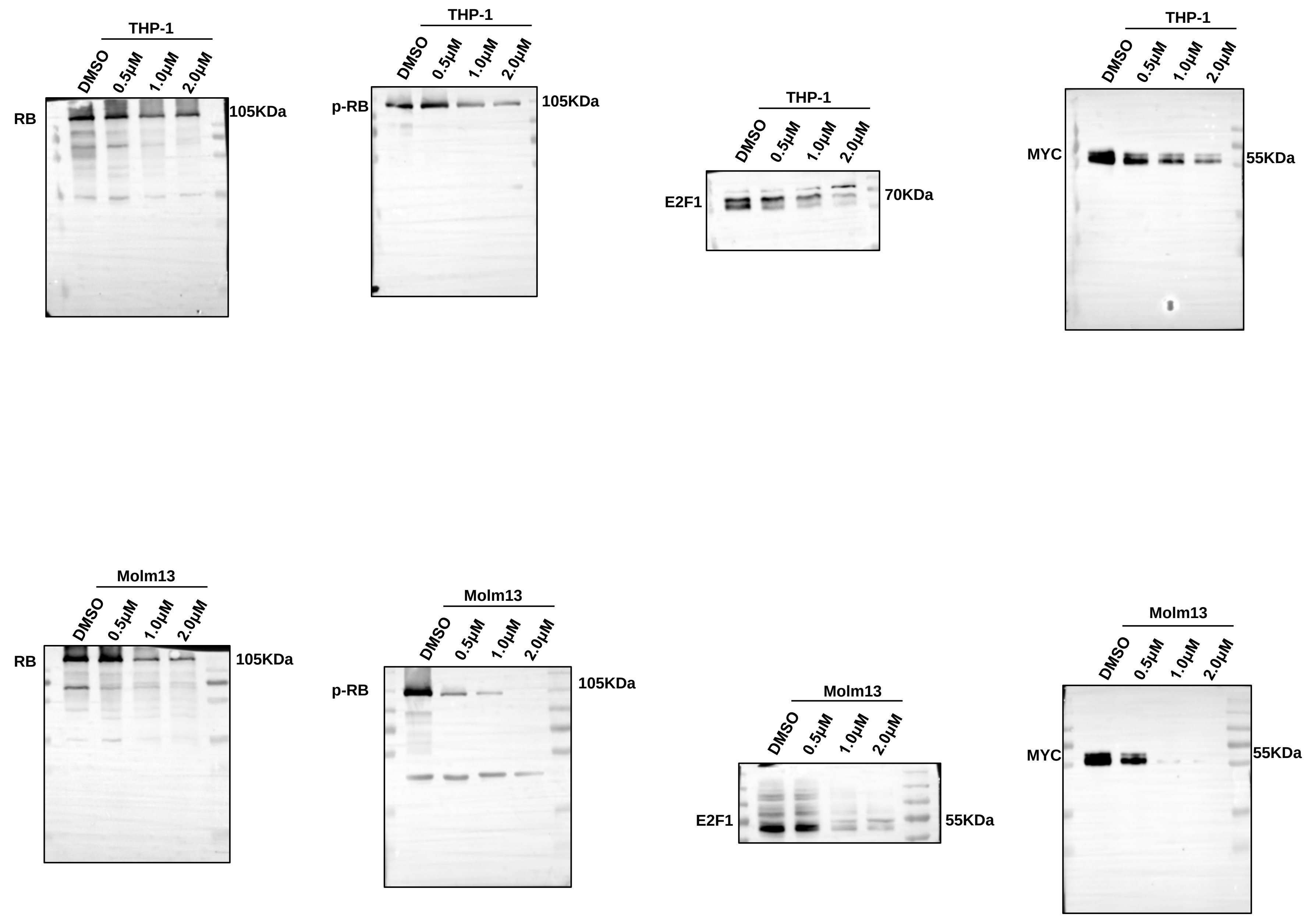

Full and uncropped western blot for Figure 4E

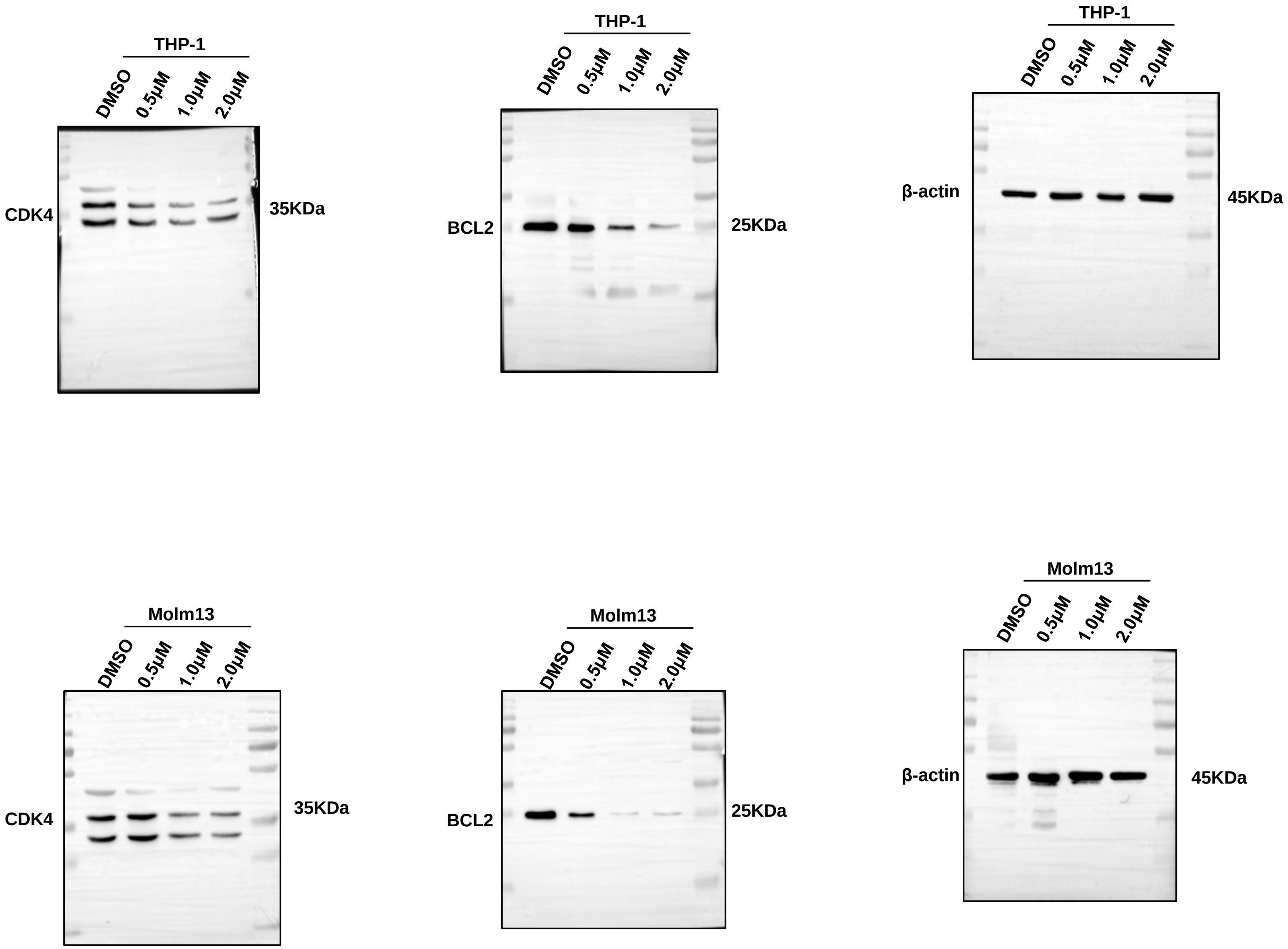

Full and uncropped western blot for Figure 5A

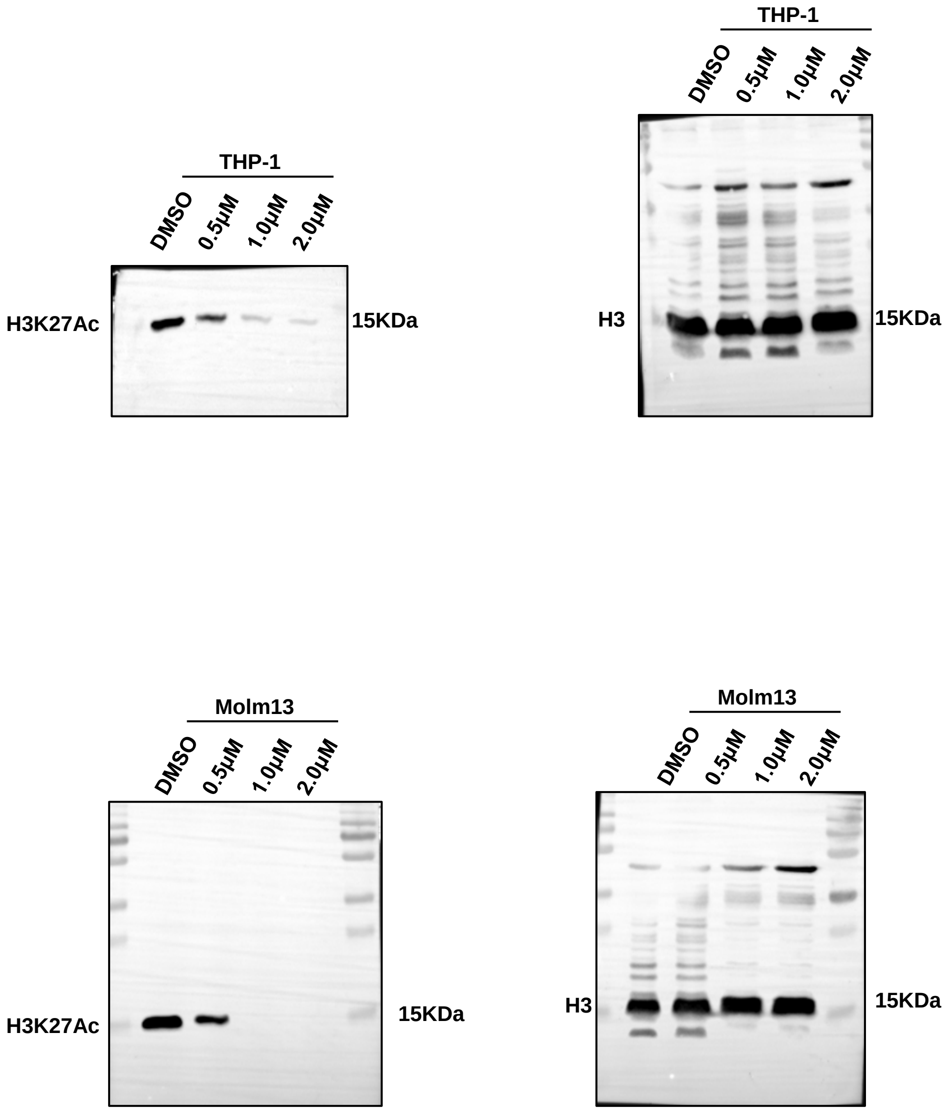

Full and uncropped western blot for Figure 5G

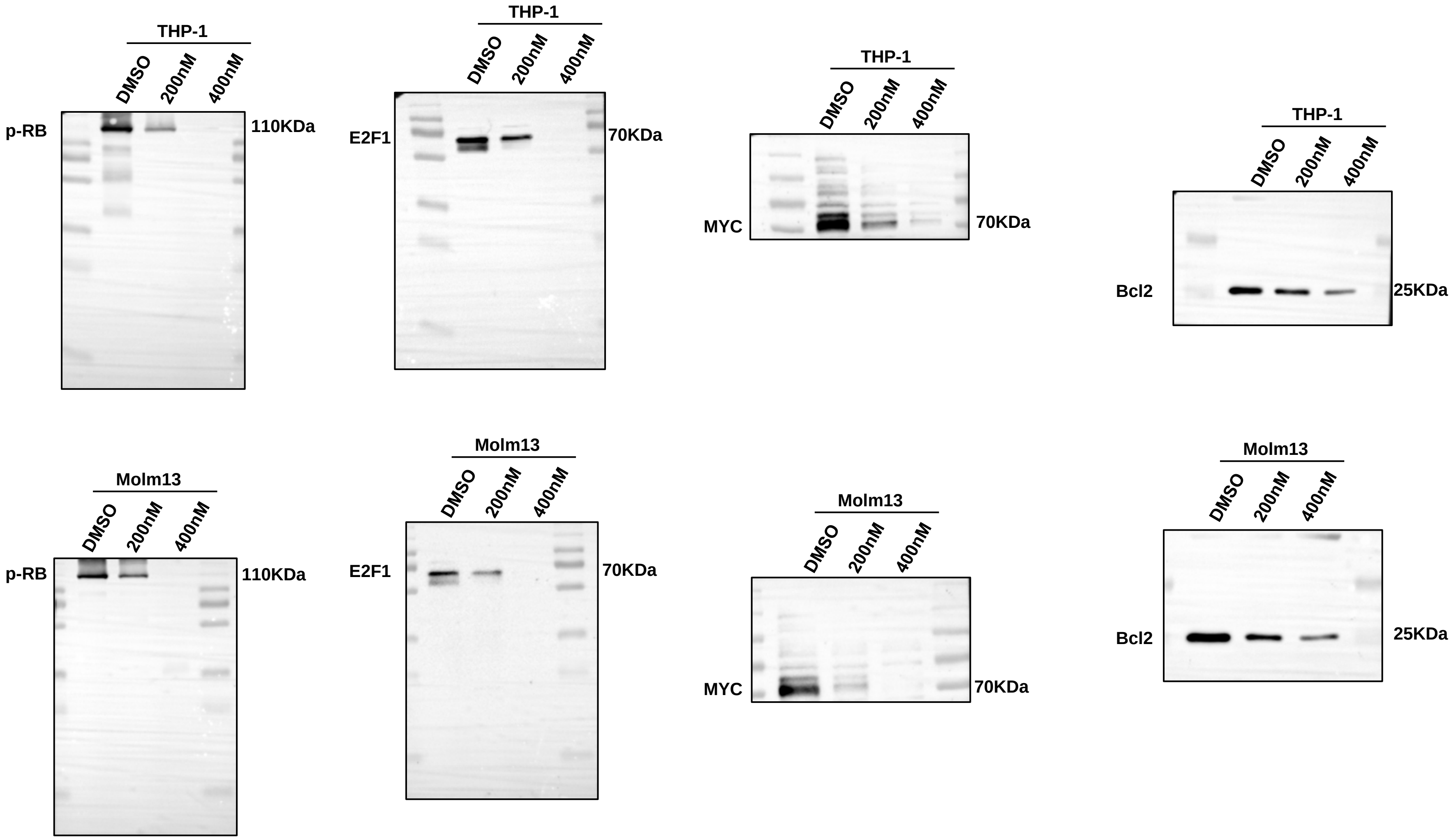

Full and uncropped western blot for Figure 5H

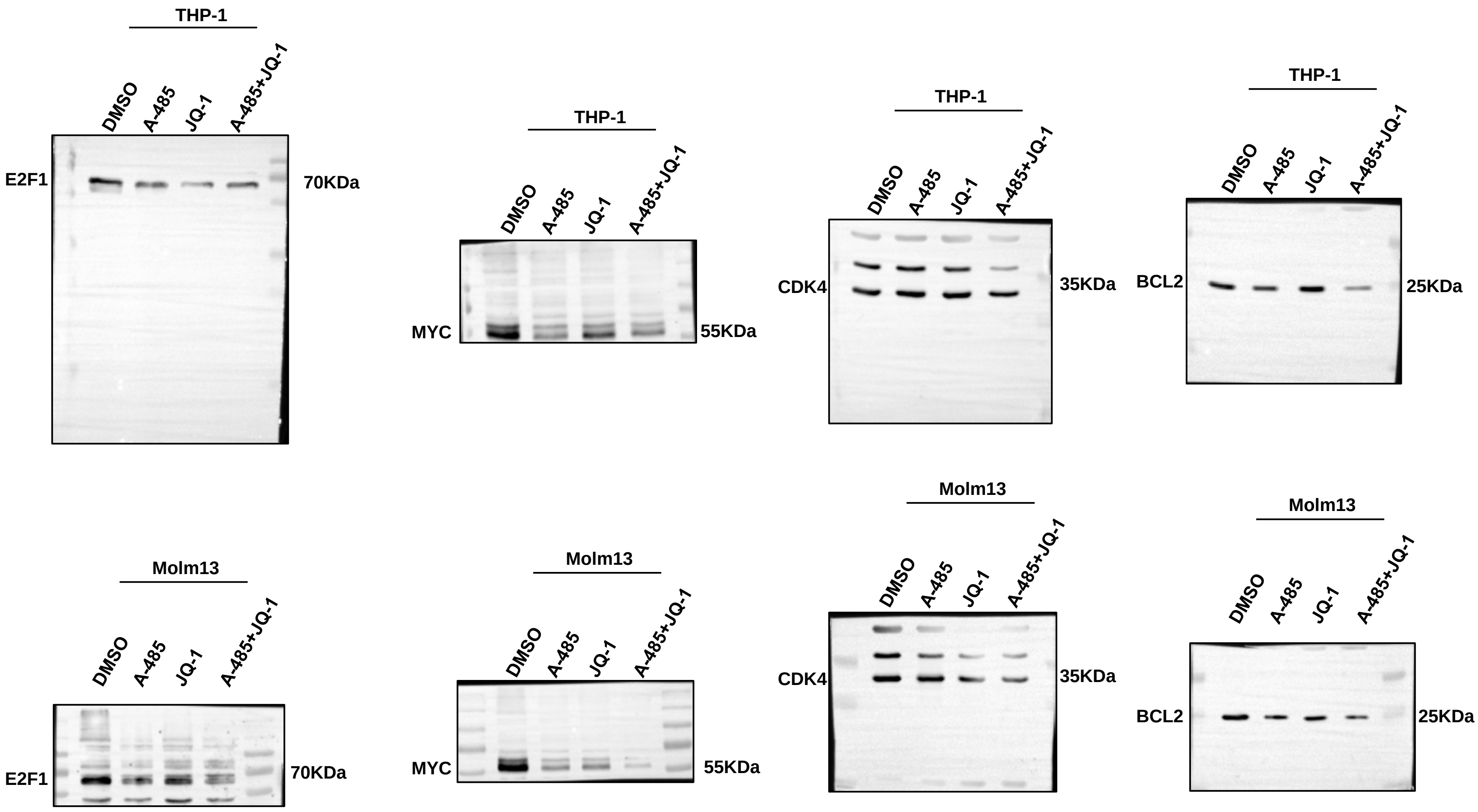

Full and uncropped western blot for Figure S6A

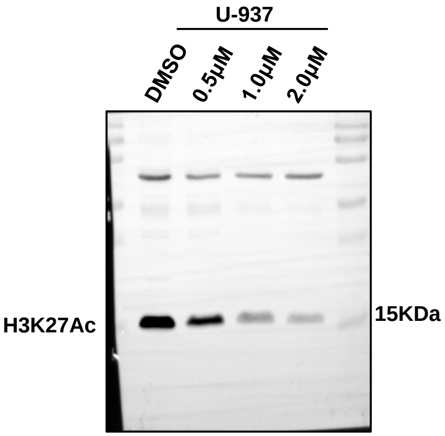

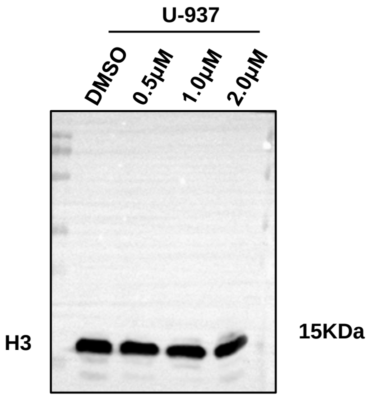

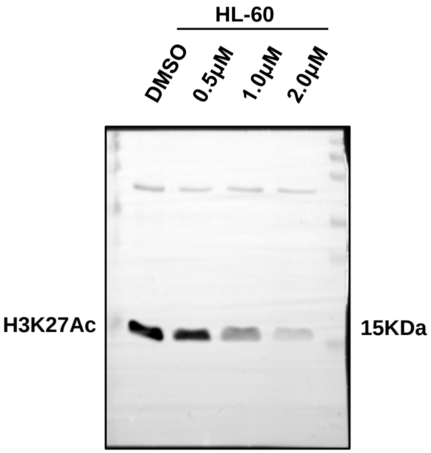

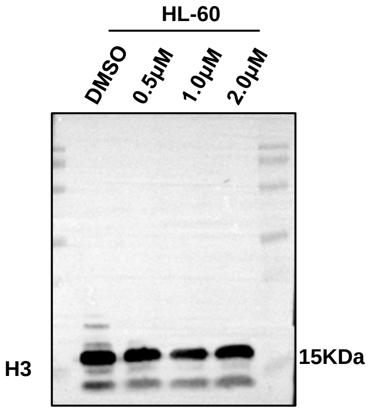

Full and uncropped western blot for Figure S6D

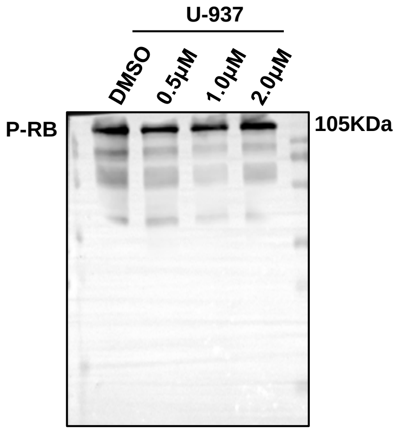

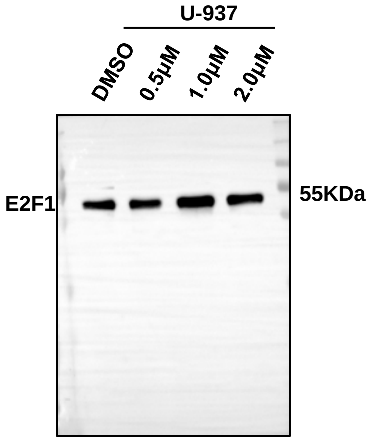

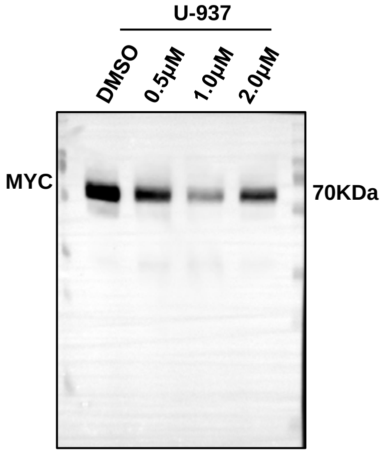

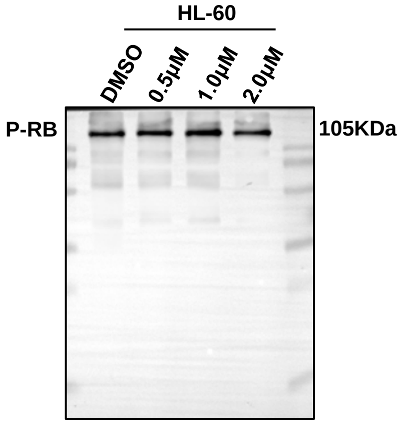

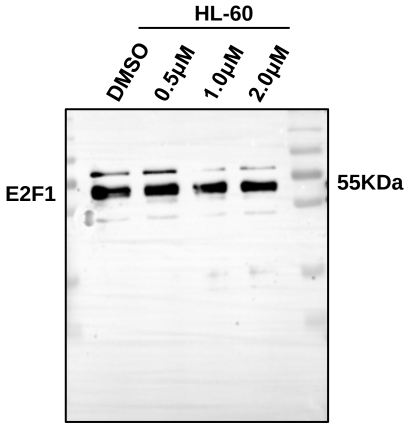

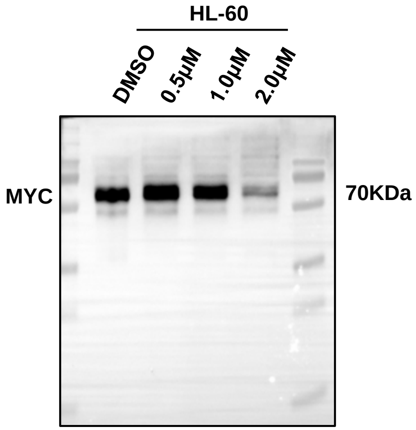

Full and uncropped western blot for Figure S6D

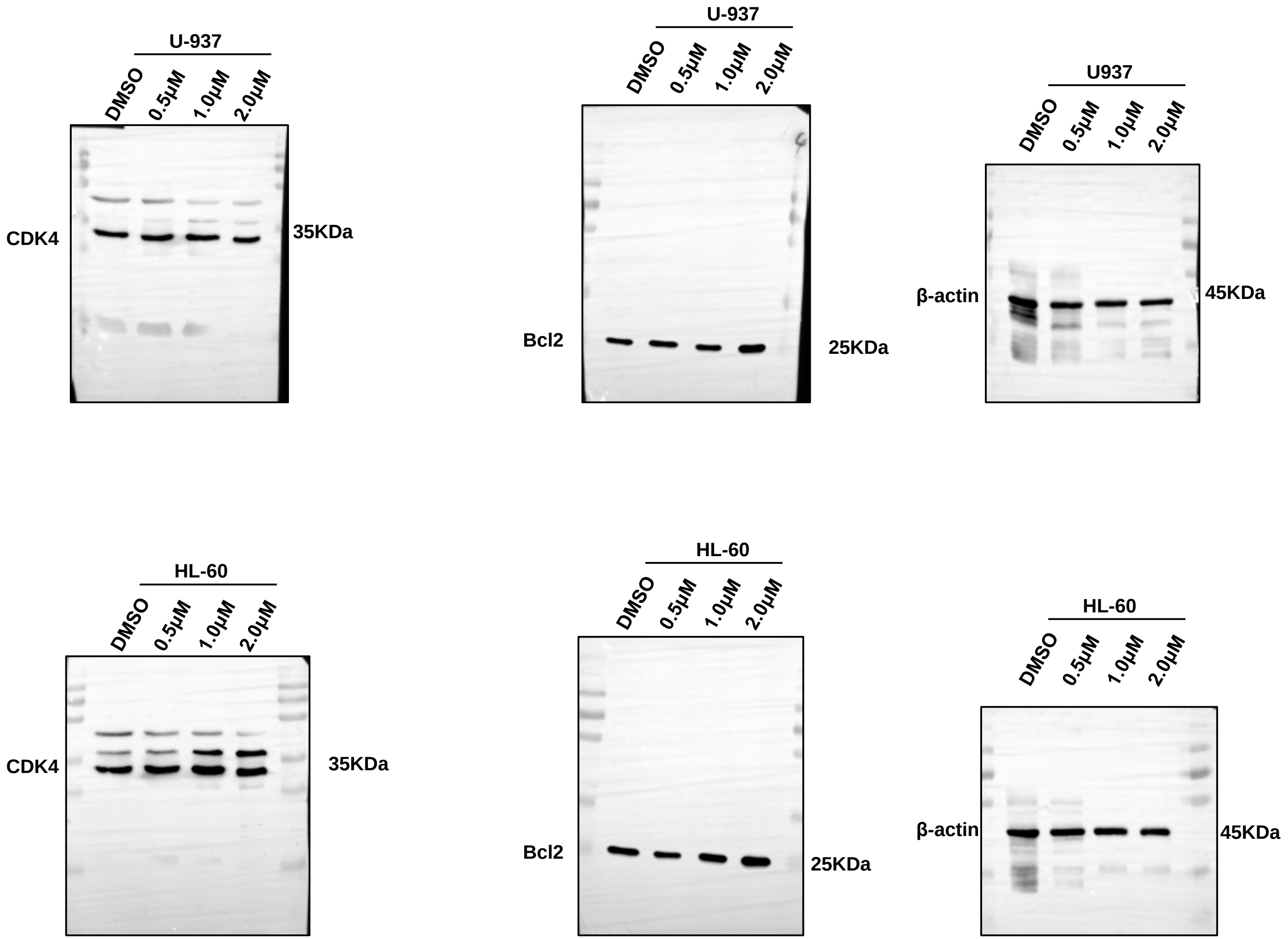

Supplement: Supplementary file 3 — Full and uncropped western blot [file 41420_2024_1940_MOESM3_ESM.pdf]
